# The association between secondhand smoke exposure and accelerated biological aging: A population-based study and Mendelian randomization analysis

**DOI:** 10.18332/tid/203865

**Published:** 2025-05-08

**Authors:** Yue Zhu, Yufan Gao, Yangguang Lu, Yukai Wang, Ziyu Pan, Huixiang Sheng, Jiajun Li, Yinuo Chen, Jialing Lou, Feng Chen, Fajing Yang

**Affiliations:** 1 The School of Public Health and Management, Wenzhou Medical University, Wenzhou, China; 2 The Second School of Medicine, Wenzhou Medical University, Wenzhou, China; 3 The First School of Medicine, School of Information and Engineering, Wenzhou Medical University, Wenzhou, China; 4 Department of Vascular Surgery, The First Affiliated Hospital of Wenzhou Medical University, Wenzhou, China

**Keywords:** secondhand smoke exposure, biological aging, diabetes, Mendelian randomization, oxidative stress

## Abstract

**INTRODUCTION:**

Aging is an irreversible biological process significantly influenced by oxidative stress, which smoking exacerbates. While the impact of direct smoking on aging is well-documented, the association between secondhand smoke (SHS) exposure and biological aging remains less explored. This study examines the connection between SHS exposure in populations and biological aging, highlighting diabetes as a potential mediator due to its established links to both SHS exposure and accelerated aging through mechanisms such as oxidative stress and chronic inflammation. It further employs genetic tools to establish a causal relationship between SHS exposure and biological aging.

**METHODS:**

This study combines secondary dataset analyses and Mendelian randomization analyses. Data from the NHANES 1999–2010 cycles were used, with serum cotinine levels indicating SHS exposure and phenotypic age, derived from age and clinical biomarkers reflecting inflammation, metabolism, and hematologic function, as the measure of biological aging. Multifactorial linear regression assessed associations, with restricted cubic splines used to explore nonlinear trends. Subgroup and mediation analyses were conducted to explore population-specific effects and the mediating role of diabetes. Two-sample Mendelian randomization (MR) using GWAS summary statistics on workplace SHS exposure (N=90168) and phenotypic age acceleration (N=6148) assessed causality.

**RESULTS:**

In the NHANES analysis, low SHS exposure was associated with a 0.37-year increase in biological aging (β=0.37; 95% CI: 0.04–0.70), while high exposure showed a 0.76-year increase (β=0.76; 95% CI: 0.23–1.29). A U-shaped association was found between log-transformed serum cotinine and biological aging (p<0.001), with a threshold at -1.53. Diabetes mediated 31.25% of this association. In the MR analysis, workplace SHS exposure was causally linked to a 3.05-year acceleration in aging (β=3.05; 95% CI: 0.24–5.85).

**CONCLUSIONS:**

SHS exposure accelerates biological aging, partly via diabetes. Genetic evidence supports a causal effect, emphasizing the need to minimize SHS exposure.

## INTRODUCTION

As the global population ages, managing the implications of aging has emerged as a paramount public health challenge^1^. Biological aging, a key factor in this challenge, has been associated with the advancement of major diseases such as cancer, diabetes, cardiovascular diseases, and neurodegenerative diseases^2^. These associations are thought to stem from cellular phenomena inherent to aging, including telomere shortening, epigenetic changes, loss of proteostasis, deregulated nutrient sensing, and mitochondrial dysfunction^3^. To accurately assess biological aging, several metrics have been developed, among which phenotypic age stands out as a robust mortality predictor, offering a more precise reflection of biological aging than chronological age alone, and thus holds significant practical utility^4^.

Smoking remains a critical global health issue, elevating the risk of cardiovascular diseases, malignancies, and overall mortality^5^. While the focus has predominantly been on active smoking, the impact of secondhand smoke (SHS) exposure has not been adequately addressed. Classified as a ‘Group 1’ carcinogen by the International Agency for Research on Cancer^6^, SHS adversely affects both adults and children, contributing to chronic obstructive pulmonary disease, stroke, and ischemic heart disease^7^. At the organ level, exposure to environmental smoke accelerates lung aging through oxidative stress responses, induces DNA damage, and stimulates autoantibody formation^8^. It also upregulates matrix metalloproteinases and reactive oxygen species production, causing premature skin aging^9^. Yet, the relationship between SHS exposure and biological aging remains underexplored, underscoring the need for epidemiological studies to further our understanding and identify potential interventions.

The link between diabetes and SHS exposure is well-established, with epidemiological evidence indicating a heightened risk of type 2 diabetes in individuals exposed to SHS, both in childhood and adulthood^10^. For those with diabetes, SHS exposure exacerbates poor glycemic control^11^. Moreover, diabetes is considered a hallmark of biological aging, potentially accelerating the aging processes^12^, suggesting that diabetes could mediate the impact of SHS exposure on biological aging.

Mendelian randomization (MR) studies, leveraging genetic variations to minimize confounding factors and reverse causality, offer a compelling approach to examining the relationship between SHS exposure and biological aging in the absence of comprehensive epidemiological evidence^13^. Therefore, this study combines a secondary analysis of data from the NHANES 1999–2010 cycles with a MR approach to explore the association between SHS exposure and biological aging, measured through phenotypic age derived from composite biomarkers. We further investigate the potential mediating role of diabetes in this association and use MR to infer a potential causal relationship between SHS exposure and biological aging.

## METHODS

### Study population

This study combines secondary dataset analyses and Mendelian randomization analyses. The National Health and Nutrition Examination Survey (NHANES) is a continuous series of nationwide surveys conducted every two years to collect representative cross-sectional data related to the health of the general US population. The NHANES program, employing a complex multi-stage probabilistic sampling design, involves household interviews, physical examinations, and laboratory tests on a selected sample of the non-institutionalized US population. Detailed study design, methods, and data collection can be found on the NHANES website. This analysis utilized publicly available data downloaded from the NHANES official website.

**Figure 1:**
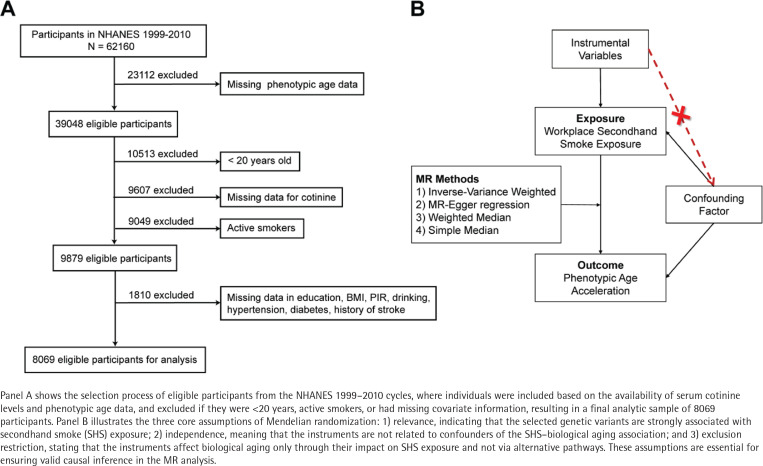
Participant selection and Mendelian randomization assumptions in the study of secondhand smoke and biological aging

This study utilized data from the NHANES 1999–2010 cycles because phenotypic age data, used to measure the level of biological aging, could only be calculated through original clinical biomarkers available in these cycles. Thus, the study included 62160 participants, excluding those missing phenotypic age data (n=23112), under 20 years of age (n=10513), missing serum cotinine levels (n=9607), active smokers (n=9049), and missing other covariates (n=1810). A final cohort of 8069 eligible participants underwent weighted analysis to represent the national sample. Figure 1 illustrates the participant selection process.

### Secondhand smoke exposure levels

To assess the level of SHS exposure, we used serum cotinine data from NHANES. Cotinine, the primary metabolite of nicotine in the body, serves as an effective biomarker for assessing smoke exposure due to its serum half-life being eight times that of nicotine^14^. Serum cotinine levels were determined using Isotope Dilution High-Performance Liquid Chromatography Atmospheric Pressure Chemical Ionization Tandem Mass Spectrometry (ID HPLC-APCI MS/MS). Detailed laboratory information can be found on the official website^15^. Active smokers, defined as those who have smoked >100 cigarettes in their lifetime^16^, were excluded to better assess SHS exposure. Based on previous studies^17^, SHS exposure levels were classified into no exposure (<0.05 ng/mL), low exposure (0.05–0.99 ng/mL), and high exposure (≥1 ng/mL).

### Measurement of biological aging

Following prior research^18^, phenotypic age was calculated using 9 biomarkers, including actual age, albumin, creatinine, glucose, C-reactive protein, lymphocyte percentage, mean cell volume, red cell distribution width, alkaline phosphatase, and white blood cell count. The calculation of phenotypic age was based on data from the NHANES III cohort and has been proven to be a reliable predictor of mortality, reflecting the state of biological aging more accurately than actual age^19^.

To further demonstrate the validity of phenotypic age, we assessed its concordance with chronological age in our study population. As shown in Supplementary file Figure 1, phenotypic age was strongly correlated with chronological age (Pearson’s r=0.92, p<2.2×10^-16^), supporting its use as a surrogate marker of biological aging.

### Covariates

Following previous research^20^, the following covariates were included: age, gender, race, education level, family poverty-income ratio, body mass index, alcohol consumption, hypertension, diabetes, and stroke history. Historical data were self-reported.

Race/ethnicity was categorized as Mexican American, Non-Hispanic White, Non-Hispanic Black, Other Hispanic, and Other (including multiracial). Education level was grouped into three categories: <high school, high school graduate, and >high school. BMI (kg/m^2^) was calculated from measured weight and height. PIR, as an indicator of socioeconomic status, was defined as the ratio of total household income to the federal poverty threshold determined by the US Census Bureau.

Smoking status was classified based on whether participants had smoked at least 100 cigarettes in their lifetime. Alcohol consumption was defined as having consumed alcohol at least 12 times in any one year. Hypertension, diabetes, and stroke were determined based on self-reported physician diagnoses; participants reporting a history of any of these conditions were classified as positive.

### Statistical analysis

Continuous variables were expressed as mean ± standard deviation (SD) or median and interquartile range (IQR), and categorical variables as frequencies (n) and percentages (%). The Kolmogorov-Smirnov test was used to assess normal distribution. Depending on the characteristics of the variables, analysis of variance, non-parametric tests, or chi-squared tests were used to compare differences between groups. Weighted multifactorial linear regression was used to evaluate the relationship between SHS exposure and biological aging, using three models: Model 1 with no adjustments; Model 2 adjusted for gender and age; and Model 3 fully adjusted for age, gender, race, education level, family poverty-income ratio, body mass index, alcohol consumption, hypertension and stroke history. Generalized additive models (GAM) assessed the nonlinear relationship between SHS exposure and biological aging; if a nonlinear relationship was observed, two-stage linear regression and recursive methods calculated the effect threshold. Restricted cubic splines visualized the relationship between SHS exposure and biological aging, with four nodes set. Due to the skewed distribution of serum cotinine levels, log transformation was necessary for enhancing the statistical efficiency of the restricted cubic splines analysis. Non-linearity was tested using a Wald test, which jointly evaluates whether the coefficients of the spline terms (excluding the linear component) are equal to zero. Stratified linear regression models conducted subgroup analyses, and likelihood ratio tests evaluated the effect across subgroups. Based on prior evidence linking SHS exposure to increased diabetes risk, and recognizing the role of diabetes in promoting biological aging, we conducted mediation analysis to examine whether diabetes mediates the effect of SHS exposure on biological aging. All statistical analyses were performed using R 4.3.0. A two-sided p<0.05 was considered statistically significant.

### Mendelian randomization

The MR study was conducted in accordance with the Strengthening the Reporting of Observational Studies in Epidemiology Using MR (STROBE-MR) guidelines^21^. Summary data from genome-wide association studies (GWAS) for SHS exposure and biological aging selected suitable SNPs as instrumental variables (IVs) for MR analysis to explore the causal relationship. The application of IVs in MR analysis relies on satisfying three critical assumptions: 1) the chosen IVs exhibit a robust association with the exposure of interest, 2) there is no confounding of the IVs with factors influencing the outcome apart from the exposure; and 3) the selected IVs solely influence the outcome through the exposure^22^. Since the workplace is a primary source of SHS exposure, data on workplace SHS exposure from the UK Biobank (GWAS ID: ukb-d-22611_1) involving 90168 participants and biological aging data from a 2021 GWAS study (GWAS ID: ebi-a-GCST90014304) with 6148 participants were analyzed. All participants were of European ancestry and provided informed consent, with no overlap between datasets.

SNPs closely associated with exposure (p<1×10^-5^) were selected, and linkage disequilibrium (LD) among SNPs was removed (r^2^<0.001, clumping distance=10000 kb). When encountering genetic variations in LD, the variant with the lowest p-value related to the exposure factor was selected. To ensure the IV’s strong association with exposure, SNPs with an F-statistic >10 were selected to exclude weak instrumental variables. The inverse-variance weighted (IVW, random effect) method served as the primary analysis method to assess the correlation between exposure and outcomes, providing accurate estimates when IVs are valid^23^. We further implemented three supplementary methods: 1) MR-Egger regression, which allows for directional pleiotropy adjustment through its intercept term; 2) Weighted Median estimator, providing valid estimates when up to 50% of instruments are invalid; and 3) Simple Median estimator as an additional pleiotropy-robust approach. Results are presented as β-values with standard errors (SE). Cochrane’s Q test evaluated heterogeneity. Potential horizontal pleiotropy was assessed using the intercept of MR-Egger regression.

## RESULTS

### Baseline characteristics according to levels of secondhand smoke exposure

The baseline characteristics categorized by different levels of SHS exposure are summarized in Table 1, with all data being appropriately weighted. The study involved 8069 participants, among whom 4947 reported no exposure to SHS, 2408 low exposure, and 714 high exposure. Notably, increased levels of SHS exposure correlated with being younger, male, of Non-Hispanic Black ethnicity, a low level of education, low family poverty-income ratio, high body mass index, and consuming alcohol (p<0.001). Furthermore, elevated exposure levels were significantly associated with hypertension (p=0.009). To illustrate biological aging more effectively, we computed both the phenotypic age and the phenotypic age residual (phenotypic age minus actual age), revealing that higher SHS exposure levels corresponded to a reduced phenotypic age and a smaller phenotypic age residual. This indicates accelerated biological aging (p<0.001) (Table 1).

**Table 1:** Baseline characteristics of participants by secondhand smoke exposure level (N=8069)

*Characteristics*	*SHS exposure level*			p*
	*Unexposed* *(N=4947)* *%*	*Low exposure* *(N=2408)* *%*	*High exposure* *(N=714)* *%*	
**Age** (years), mean ± SD	46.99 ± 16.72	42.51 ± 16.22	39.24 ± 15.58	<0.001
**Male**	34.45	44.98	61.90	<0.001
**Race**				<0.001
Mexican American	26.36	19.10	9.24	
Other Hispanic	6.59	5.98	4.62	
Non-Hispanic White	49.55	41.53	43.42	
Non-Hispanic Black	12.92	28.74	39.08	
Other	4.59	4.65	3.64	
**Education level**				<0.001
<High school	23.91	27.78	28.71	
High school	18.82	24.04	28.85	
>High school	57.27	48.17	42.44	
**Family PIR**, median (IQR)	2.71 (1.36–4.85)	2.16 (1.12–3.96)	1.76 (1.00–3.40)	<0.001
**Health status**				
BMI (kg/m^2^), mean ± SD	28.29 ± 6.42	29.28 ± 6.83	29.58 ± 7.71	<0.001
Drinking	55.75	60.59	76.61	<0.001
Hypertension	31.96	29.82	26.89	0.009
Diabetes	9.74	9.30	9.38	0.830
History of stroke	2.93	2.37	2.38	0.343
Phenotypic age (years), mean ± SD	41.36 ± 18.63	37.82 ± 18.61	35.28 ± 19.19	<0.001
Phenotypic age residual, median (IQR)	-6.03 (-9.38 – -1.66)	-5.06 (-8.50 – -0.71)	-4.54 (-8.11– -0.36)	<0.001

Characteristics of the study population stratified by serum cotinine-defined secondhand smoke (SHS) exposure: unexposed (<0.05 ng/mL), low exposure (0.05–0.99 ng/mL), and high exposure (≥1 ng/mL). All estimates were weighted according to NHANES sampling design.

* Calculated using ANOVA, Kruskal-Wallis, or chi-square tests where appropriate.

PIR: poverty-income ratio. BMI: body mass index. IQR: interquartile range.

### Multifactorial linear regression analysis

We conducted weighted multifactorial linear regression analysis to investigate the relationship between SHS exposure and biological aging, employing three different models for this analysis. Model 1 applied no adjustments; Model 2 adjusted for gender and age; and Model 3 was fully adjusted for all covariates. As depicted in Table 2, Model 1 (unadjusted) showed that, compared to individuals with no exposure, low exposure to SHS was associated with a 0.95-year acceleration in biological aging (β=0.95; 95% CI: 0.59–1.31), and high exposure resulted in a 1.68-year increase (β=1.68; 95% CI: 1.11–2.24). Model 2, adjusting for gender and age, indicated that low exposure accelerated biological aging by 1.07 years (β=1.07, 95% CI: 0.71–1.44), and high exposure by 1.85 years (β=1.85; 95% CI: 1.28–2.42). In the fully adjusted Model 3, low exposure was linked to a 0.37-year acceleration in biological aging (β=0.37; 95% CI: 0.04–0.70), and high exposure by 0.76 years (β=0.76; 95% CI: 0.23–1.29) (Table 2). To further test the robustness of our findings, we conducted a sensitivity analysis by additionally adjusting for physical activity, given its importance as a potential covariate of interest. The results remained consistent: compared with the non-exposed group, low SHS exposure was associated with a 0.37-year increase (β=0.37; 95% CI: 0.04–0.70) in biological aging, and high exposure with a 0.74-year increase (β=0.74; 95% CI: 0.21–1.27) (Supplementary file Table 1), confirming the stability of our main findings.

**Table 2:** Multivariable linear regression models assessing the association between SHS exposure and biological aging

	*Model 1* *β (95% CI)*	*p*	*Model 2* *β (95% CI)*	*p*	*Model 3* *β (95% CI)*	*p*
Unexposed ®						
Low exposure	0.95 (0.59–1.31)	<0.001	1.07 (0.71–1.44)	<0.001	0.37 (0.04–0.70)	0.030
High exposure	1.68 (1.11–2.24)	<0.001	1.85 (1.28–2.42)	<0.001	0.76 (0.23–1.29)	0.005

SHS exposure based on serum cotinine concentration: no exposure (<0.05 ng/mL), low exposure (0.05–0.99 ng/mL), and high exposure (≥1 ng/mL). Model 1: no adjustments. Model 2: adjusted for gender and age. Model 3: fully adjusted for age, gender, race, education level, family poverty-income ratio, body mass index, alcohol consumption, hypertension and stroke history. Beta coefficients (β) and 95% confidence intervals (CI) representing the association between SHS exposure categories and phenotypic age (years), using weighted linear regression. All models were weighted using NHANES survey sample weights. ® Reference category.

### Threshold effects and non-linear associations

**Figure 2:**
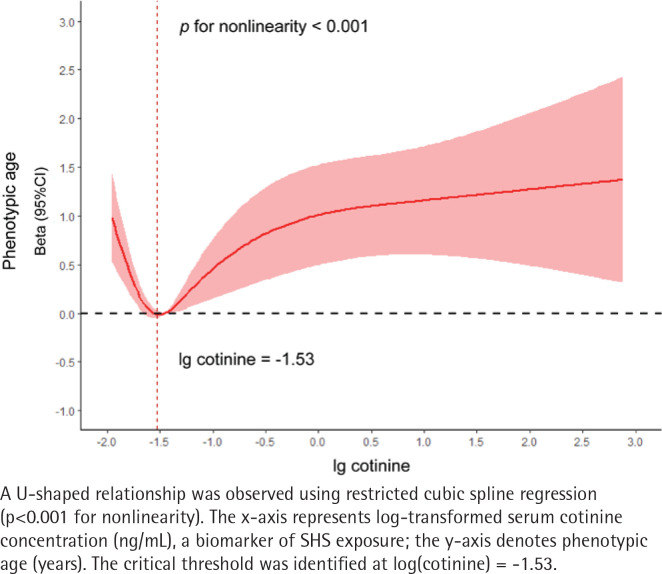
Nonlinear association between log-transformed serum cotinine and biological aging

To further examine the non-linear association between SHS exposure and biological aging, we utilized a generalized additive model (GAM) for two-stage linear regression and applied recursive methods for determining the effect threshold^24^. Employing restricted cubic splines, we examined the relationship between log-transformed serum cotinine concentrations (a proxy for SHS exposure) and biological aging. The results revealed a U-shaped relationship between log serum cotinine concentration and biological aging (p for nonlinearity <0.001), with a log-critical threshold identified at -1.53 (Figure 2). Two-stage linear regression showed that, for log serum cotinine concentrations below -1.53, each unit increase in the log-transformed cotinine concentration was associated with a 1.89-year delay in biological aging (β= -1.89; 95% CI: -2.79 – -1.00); conversely, above -1.53, each unit increase was linked to a 0.38-year acceleration in aging (β=0.38; 95% CI: 0.20–0.57) (Supplementary file Table 2).

### Subgroup and mediation analyses

In subgroup analyses that included variables such as age, gender, ethnicity, education level, BMI, hypertension, diabetes, and alcohol consumption, we found that low exposure to SHS remained significantly associated with biological aging in males, Non-Hispanic Whites, individuals with a high school education, those with a BMI <30 kg/m^2^, and alcohol consumers. High exposure was significantly linked to biological aging in individuals under 60 years, females, Non-Hispanic Whites, those with an education level beyond high school, individuals with a BMI <25 kg/m^2^, and those with hypertension, diabetes, and alcohol consumption habits. Moreover, significant interactions were observed for age (p for interaction=0.023), gender (p for interaction=0.011), and diabetes status (p for interaction=0.014), indicating that the association between SHS exposure and biological aging may be modified by these factors (Supplementary file Table 3).

**Figure 3:**
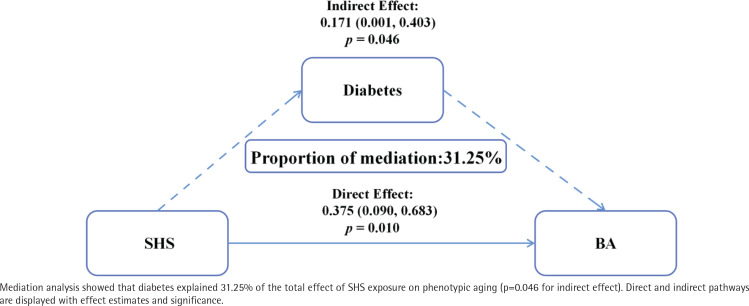
Mediation effect of diabetes in the association between SHS exposure and biological aging

Through subgroup analysis, we observed that the association between SHS exposure and biological aging was amplified among individuals with diabetes (Supplementary file Table 3). Therefore, we used a mediation effect model to explore whether the association between SHS exposure and biological aging was mediated by diabetes. After adjusting for all covariates and applying weighting, results indicated a significant direct effect of SHS exposure on biological aging (β=0.38; 95% CI: 0.09–0.68, p=0.010) and an indirect effect through the impact of SHS exposure levels on diabetes, ultimately affecting biological aging (β=0.17; 95% CI: 0.00–0.40, p=0.046). The mediation proportion attributed to diabetes was 31.25%, establishing a partial mediation model (Figure 3).

### Mendelian randomization analysis

**Figure 4:**
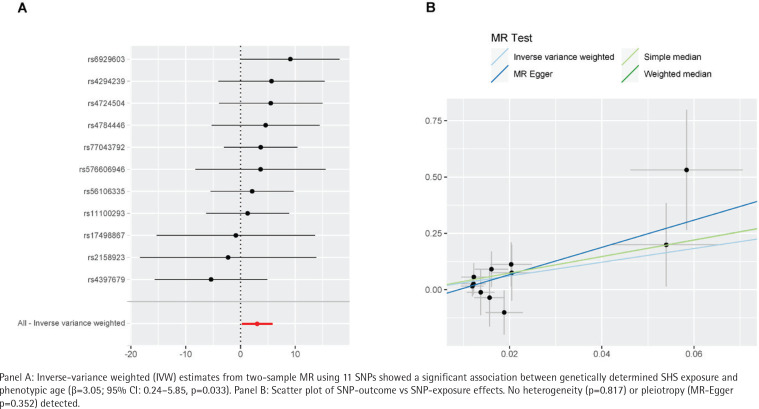
Mendelian randomization analysis of workplace SHS exposure and biological aging

In our MR analysis, we specifically focused on workplace SHS exposure as it represents a major source of environmental smoke exposure for adults. While residential exposure constitutes another important source of SHS, our MR design intentionally utilized genetic variants associated with workplace exposure to ensure biological plausibility of the instrumental variables and minimize confounding from shared household environments. It should be emphasized that this MR analysis specifically addresses workplace exposure. The IVW model revealed that genetically predicted workplace SHS exposure was associated with a 3.05-year acceleration in biological aging (β=3.05; 95% CI: 0.24–5.85, p=0.033) (Figure 4A). The scatter plot shows that the results of merging MR models such as MR Egger, Simple Median, and Weighted Median are basically consistent, indicating the robustness of the results (Figure 4B). MR-Egger analysis showed no horizontal pleiotropy (intercept p=0.352), though this method requires satisfaction of the Instrument Strength Independent of Direct Effect (InSIDE) assumption, and that the strength of SNP-exposure associations is independent of their pleiotropic effects. Cochrane’s Q test indicated no heterogeneity (p=0.817), supporting the robustness of these workplace-specific findings.

## DISCUSSION

This study represents a pioneering analysis of the relationship between SHS exposure and biological aging. Utilizing weighted data representative of the US population, our findings demonstrate that increased levels of SHS exposure are associated with accelerated biological phenotypic aging. Specifically, our analysis reveals that low exposure to SHS results in a 0.37-year acceleration in biological aging, whereas high exposure leads to a 0.76-year acceleration. Notably, we observed a U-shaped association between log-transformed serum cotinine levels and biological aging, identifying a log-critical threshold of -1.53. Additionally, mediation analysis indicates that diabetes serves as a mediator in the linkage between SHS exposure and biological aging, accounting for 31.25% of the effect. Genetically determined exposure to SHS in the workplace is correlated with a 3.05-year acceleration in phenotypic age.

The detrimental effects of tobacco smoke exposure on the progression of various diseases are well-documented^25^. Previous research has established that smokers exhibit shorter telomere lengths, with a negative correlation between the duration of smoking and telomere length, highlighting the association between tobacco smoke exposure and age-related diseases^26^. Moreover, prenatal exposure to smoke has been closely linked to DNA methylation changes in offspring^27^. However, these studies have not distinguished between active smokers and passive exposure, thereby not adequately demonstrating the specific impact of SHS on the acceleration of biological aging. The association of SHS exposure with diseases such as headache, heart failure, and cancer are well established^25^, yet its link with biological aging has remained uncertain.

Biological aging is quantifiable through various methodologies, including the Klemera-Doubal method (KDM)^28^, phenotypic age (PA)^18^, and DNA methylation age. These approaches, which integrate clinical biomarkers and genetic data, offer a more precise reflection of an individual’s biological aging than chronological age alone. In this study, we employed data of phenotypic age, known for its clinical applicability and ease of acquisition. The calculation of phenotypic age, based on NHANES III population data, has been validated as a reliable mortality predictor, more accurately depicting biological aging^19^. Accelerated biological aging, as measured by phenotypic age, is associated with increased risks of depression, anxiety, cancer, and rheumatoid arthritis, underscoring its utility in aging research^29^.

The link between SHS exposure and biological aging is potentially mediated by the generation of free radicals, which induce oxidative stress and inflammation^30^, contributing to telomere shortening, diminished DNA repair capacity^31^, and ultimately, cellular aging and apoptosis. In the context of lung aging related to smoking, the increased peripheral lung expression of 8-hydroxy-2’-deoxyguanosine in smokers may reflect oxidative stress from smoking^32^. Workplace SHS exposure has been associated with an elevated diabetes risk among workers^33^, linking passive and active smoking with type 2 diabetes risk in women^34^. Given the complex interactions between diabetes and biological aging^12^, diabetes likely plays a significant role in the pathway connecting SHS exposure to biological aging.

Serum cotinine levels are a reliable marker for assessing SHS exposure^17^, offering higher accuracy than self-reported measures by minimizing recall bias^35^. Our findings of a U-shaped association between log-transformed serum cotinine concentration and biological aging, with a log-critical threshold of -1.53, suggest that very low levels of serum cotinine may also accelerate aging. Nonetheless, this does not imply that absence of SHS exposure accelerates aging. Cotinine itself possesses biological activities, including antipsychotic, anti-anxiety, and antidepressant effects^36^, and influences serotonergic, cholinergic, and dopaminergic systems, exhibiting anti-inflammatory properties^37^. Identifying the serum cotinine concentration threshold elucidates cotinine’s biological roles in aging and aids in further segmenting populations exposed to SHS.

### Strengths and limitations

The strengths of this study lie in its novel exploration of SHS exposure’s effect on biological aging, unveiling an association with accelerated aging and providing fresh epidemiological insights for reducing SHS exposure and delaying aging. The use of nationally representative data with a substantial sample size strengthens our conclusions. By analyzing the threshold effect, we determined a U-shaped relationship between serum cotinine concentration and biological aging, establishing an optimal concentration for aging delay. This finding lays down the groundwork for mechanistic studies and further population segmentation. Particularly, our analysis highlights the relevance of this relationship in the diabetic population. Through two-sample MR, we corroborated the causal link between workplace SHS exposure and biological aging, supplementing cross-sectional studies with genetic evidence.

However, this study’s limitations include the inability to account for all potential covariates due to data constraints and to explore alternative biological aging measures comprehensively. Our MR analysis also has several limitations. First, the Mendelian randomization analysis relied on genetic instruments derived from European ancestry GWAS datasets for both workplace SHS exposure and phenotypic age acceleration. While we minimized weak instrument bias by selecting SNPs with strong associations and performed sensitivity analyses to address pleiotropy, the lack of multi-ethnic GWAS summary statistics limits the generalizability of our causal estimates to non-European populations. Differences in linkage disequilibrium patterns, allele frequencies, and gene-environment interactions across ancestries may influence the validity of genetic instruments in diverse groups. Second, sex-stratified analyses were precluded by the absence of sex-specific GWAS data for phenotypic age acceleration. Third, while MR-Egger regression and Cochran’s Q test indicated no significant horizontal pleiotropy or heterogeneity, residual confounding from unmeasured pleiotropic pathways cannot be entirely ruled out. These limitations underscore the need for future large-scale GWAS efforts in underrepresented populations to strengthen causal inference and improve the external validity of MR findings in aging research.

## CONCLUSIONS

Our investigation conclusively links SHS exposure to accelerated biological aging, a relationship that is partially mediated by the presence of diabetes. Notably, we identified a U-shaped association between log-transformed serum cotinine concentrations and biological aging, suggesting nuanced effects at different exposure levels. Further bolstering our findings, genetic evidence corroborates the acceleration of biological aging due to SHS exposure, specifically in workplace environments. These insights collectively underscore the potential public health benefit of reducing SHS exposure as a viable strategy to decelerate the process of biological aging. Implementing measures to minimize SHS exposure could play a crucial role in extending a healthy life span and reducing the incidence of age-related diseases, thereby improving overall population health.

## Supplementary Material



## Data Availability

The data supporting this research are available from: https://wwwn.cdc.gov/nchs/nhanes/Default.aspx (Accessed: 5 October 2024).
