# Supplementary material for: The association between secondhand smoke exposure and accelerated biological aging: A population-based study and Mendelian randomization analysis
Source: Tob Induc Dis. 2025 May 8;23:10.18332/tid/203865. doi: 10.18332/tid/203865 (PMC12060151; doi:10.18332/tid/203865)
Supplement: Supplementary file 1 [file TID-23-59-s1.pdf]

SUPPLEMENTARY MATERIALS

The Association Between Secondhand Smoke Exposure and Accelerated Biological Aging: A Population-Based Study and Mendelian Randomization Analysis

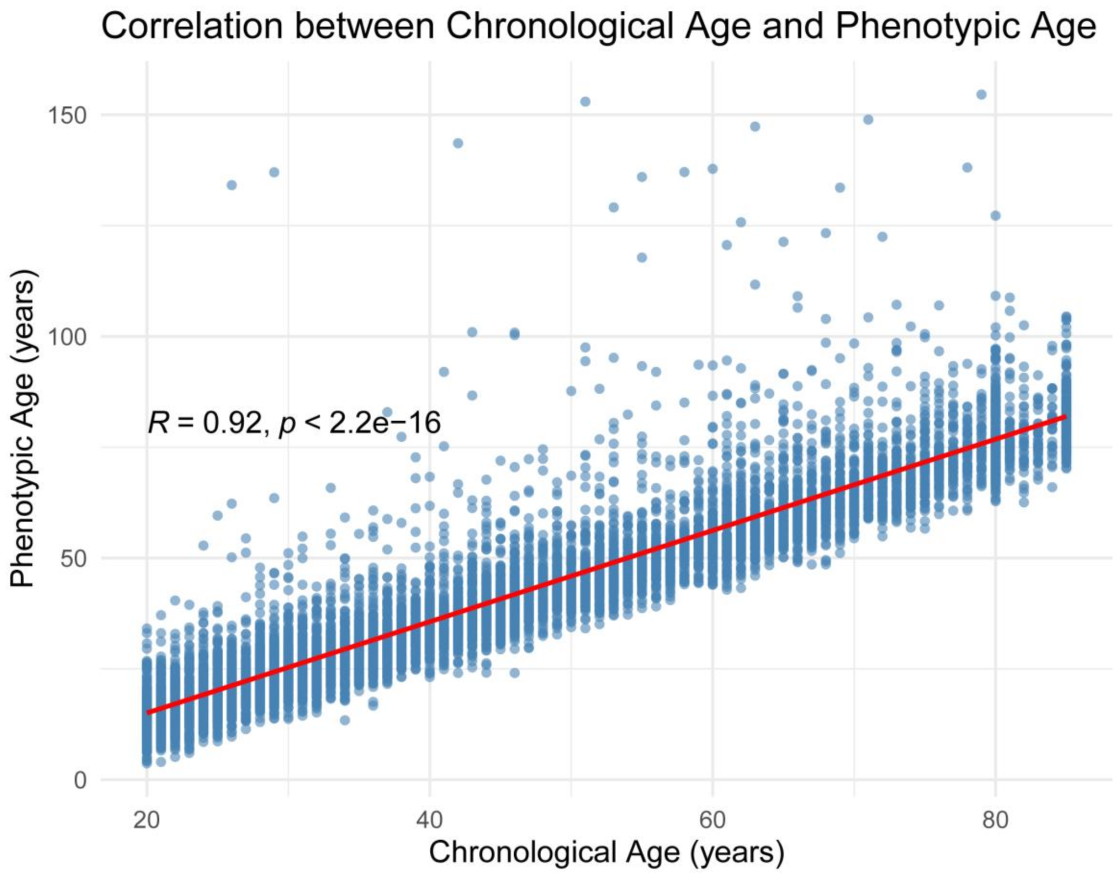

Supplementary Figure 1. Correlation analysis between chronological age and phenotypic age.

Legend: This scatter plot illustrates the correlation between chronological age and phenotypic age among study participants. Each dot represents an individual observation. The solid line indicates the fitted regression line, while the shaded area represents the 95% confidence interval. A strong positive correlation was observed (Pearson's r = 0.92, p < 0.001), indicating that phenotypic age closely tracks chronological age while capturing additional biological variation.

Supplementary Table 1. Sensitivity analysis of the association between secondhand smoke exposure and phenotypic age.

	**Model 1**<br>**β (95%CI)**	***p*-value**	**Model 2**<br>**β (95%CI)**	***p*-value**	**Model 3**<br>**β (95%CI)**	***p*-value**
Unexposed	Ref		Ref		Ref	
Low exposure	0.95 (0.59, 1.31)	<0.001	1.07 (0.71, 1.44)	<0.001	0.37 (0.04, 0.70)	0.030
Heavy exposure	1.68 (1.11, 2.24)	<0.001	1.85 (1.28, 2.42)	<0.001	0.74 (0.21, 1.27)	0.006

Note: Secondhand smoke exposure was categorized as follows: no exposure (< 0.05 ng/mL), light exposure (0.05–0.99 ng/mL), and heavy exposure (≥ 1 ng/mL), based on serum cotinine concentrations. All models were weighted using NHANES survey sample weights.

Model 1: no adjustments

Model 2: adjusted for gender and age

Model 3: fully adjusted for age, gender, race, educational level, family poverty-income ratio, body mass index, alcohol consumption, hypertension, stroke history and physical activity.

Legend: This table presents the results of a sensitivity analysis evaluating the association between secondhand smoke (SHS) exposure and

phenotypic age, accounting for physical activity. SHS exposure was categorized into unexposed (< 0.05 ng/mL), low exposure (0.05–0.99 ng/mL), and heavy exposure (≥ 1 ng/mL) based on serum cotinine concentrations. Three models were used: Model 1 (unadjusted), Model 2 (adjusted for gender and age), and Model 3 (fully adjusted for age, gender, race, educational level, family poverty-income ratio, body mass index, alcohol consumption, hypertension, stroke history, and physical activity). All estimates were weighted using NHANES survey sample weights. Phenotypic age was significantly higher in both low and heavy SHS exposure groups compared to the unexposed group across all models.

Supplementary Table 2. Piecewise linear regression of log-transformed cotinine and biological aging acceleration.

	**PhenoAge acceleration**	
	β (95% CI)	*p*-value
Fitting model by standard regression	0.17 (0.01, 0.33)	0.040
Fitting model by two-piecewise regression		
Inflection point of lg (cotinine) (K)	-1.53	
<K	-1.89 (-2.79, -1.00)	< 0.001
≥K	0.38 (0.20, 0.57)	< 0.001
p for log likelihood ratio test		< 0.001

Legend: Results of two-piecewise linear regression models assessing the nonlinear association between log-transformed serum cotinine concentrations and biological aging acceleration. A threshold was identified at log(cotinine) = -1.53. Below the threshold, log cotinine was associated with delayed aging, while above the threshold, it was associated with accelerated aging. Results are presented as β (95% CI). *p*-value for the log-likelihood ratio test indicates model fit improvement by using a piecewise approach. All models were weighted using NHANES survey sample weights.

Supplementary Table 3. Subgroup analysis of the association between secondhand smoke exposure and biological aging

Subgroups	Low exposure β (95% CI)	*p* value	Heavy exposure β (95% CI)	*p* value	*p* for interaction
Age, year					0.023
< 60	0.299 (−0.074, 0.671)	0.116	0.795 (0.217, 1.373)	0.007	
≥ 60	0.584 (−0.147, 1.315)	0.118	0.101 (−1.273, 1.476)	0.885	
Gender (Male)					0.011
Yes	0.788 (0.310, 1.267)	0.001	0.262 (−0.407, 0.932)	0.442	
No	0.043 (−0.411, 0.498)	0.851	1.793 (0.944, 2.641)	< 0.001	
Race					0.512
Mexican American	0.626 (−0.086, 1.337)	0.085	0.417 (−1.103, 1.937)	0.591	
Other Hispanic	−0.261 (−1.790, 1.269)	0.738	0.892 (−1.618, 3.402)	0.487	
Non−Hispanic White	0.520 (0.085, 0.956)	0.019	0.935 (0.233, 1.638)	0.009	
Non−Hispanic Black	0.108 (−0.870, 1.086)	0.828	0.944 (−0.385, 2.273)	0.164	
Other races	0.188 (−1.772, 2.148)	0.851	0.382 (−2.986, 3.750)	0.824	
Education level					0.713
< high school	0.436 (−0.324, 1.197)	0.261	0.084 (−1.079, 1.247)	0.888	
High school	0.955 (0.251, 1.659)	0.008	0.041 (−0.992, 1.073)	0.938	
> high school	0.110 (−0.331, 0.551)	0.625	1.383 (0.639, 2.127)	< 0.001	
BMI, kg/m2					0.275
< 25	0.591 (0.044, 1.138)	0.034	1.691 (0.766, 2.616)	< 0.001	
25−30	0.675 (0.084, 1.266)	0.025	0.765 (−0.159, 1.689)	0.105	
≥ 30	−0.001 (−0.595, 0.592)	0.997	0.207 (−0.716, 1.129)	0.661	
Hypertension					0.177
Yes	0.622 (−0.173, 1.416)	0.125	1.544 (0.240, 2.848)	0.020	
No	0.264 (−0.075, 0.603)	0.127	0.469 (−0.067, 1.005)	0.087	
Diabetes					0.014
Yes	1.578 (−0.745, 3.901)	0.184	6.240 (2.799, 9.680)	< 0.001	
No	0.244 (−0.054, 0.542)	0.109	0.224 (−0.255, 0.702)	0.359	
Drinking					0.425
Yes	0.672 (0.260, 1.084)	0.001	1.016 (0.410, 1.622)	0.001	
No	−0.137 (−0.699, 0.426)	0.634	0.175 (−0.940, 1.289)	0.759	

Low exposure

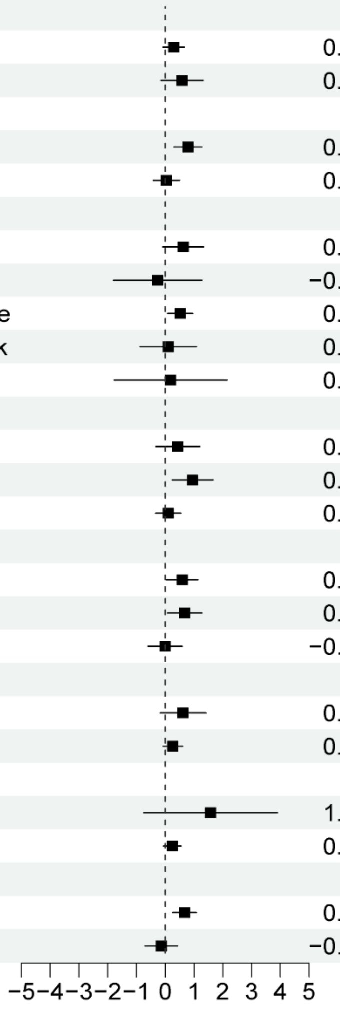

Heavy exposure

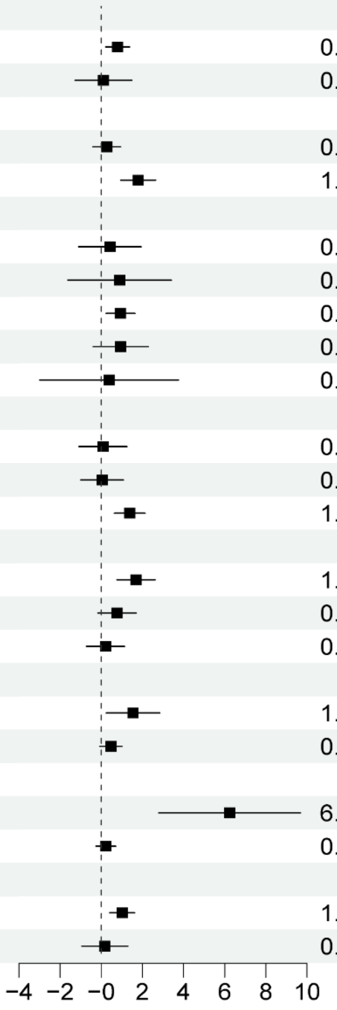

Legend: Weighted linear regression was stratified by sex, age, race, education, BMI, hypertension, diabetes, and alcohol use. The plot shows estimated beta coefficients and 95% confidence intervals for light and heavy SHS exposure versus no exposure.

© 2025 Zhu Y. et al.
